# Microbial characterization of the nasal cavity in patients with allergic rhinitis and non-allergic rhinitis

**DOI:** 10.3389/fcimb.2023.1166389

**Published:** 2023-04-25

**Authors:** Yanlu Che, Nan Wang, Qianzi Ma, Junjie Liu, Zhaonan Xu, Qiuying Li, Jingting Wang, Yanan Sun

**Affiliations:** Department of Otorhinolaryngology, Head and Neck Surgery, The Second Affiliated Hospital of Harbin Medical University, Harbin, China

**Keywords:** allergic rhinitis, non-allergic rhinitis, microecology, 16SrDNA, macrogenome

## Abstract

**Introduction:**

Although recent studies have shown that the human microbiome is involved in the pathogenesis of allergic diseases, the impact of microbiota on allergic rhinitis (AR) and non-allergic rhinitis (nAR) has not been elucidated. The aim of this study was to investigate the differences in the composition of the nasal flora in patients with AR and nAR and their role in the pathogenesis.

**Method:**

From February to September 2022, 35 AR patients and 35 nAR patients admitted to Harbin Medical University’s Second Affiliated Hospital, as well as 20 healthy subjects who underwent physical examination during the same period, were subjected to 16SrDNA and metagenomic sequencing of nasal flora.

**Results:**

The microbiota composition of the three groups of study subjects differs significantly. The relative abundance of Vibrio vulnificus and Acinetobacter baumanni in the nasal cavity of AR patients was significantly higher when compared to nAR patients, while the relative abundance of Lactobacillus murinus, Lactobacillus iners, Proteobacteria, Pseudomonadales, and Escherichia coli was lower. In addition, Lactobacillus murinus and Lacttobacillus kunkeei were also negatively correlated with IgE, while Lacttobacillus kunkeei was positively correlated with age. The relative distribution of Faecalibacterium was higher in moderate than in severe AR patients. According to KEGG functional enrichment annotation, ICMT(protein-S-isoprenylcysteine O-methyltransferase,ICMT) is an AR microbiota-specific enzyme that plays a role, while glycan biosynthesis and metabolism are more active in AR microbiota. For AR, the model containing Parabacteroides goldstemii, Sutterella-SP-6FBBBBH3, Pseudoalteromonas luteoviolacea, Lachnospiraceae bacterium-615, and Bacteroides coprocola had the highest the area under the curve (AUC), which was 0.9733(95%CI:0.926-1.000) in the constructed random forest prediction model. The largest AUC for nAR is 0.984(95%CI:0.949−1.000) for the model containing Pseudomonas-SP-LTJR-52, Lachnospiraceae bacterium-615, Prevotella corporis, Anaerococcus vaginalis, and Roseburia inulinivorans.

**Conclusion:**

In conclusion, patients with AR and nAR had significantly different microbiota profiles compared to healthy controls. The results suggest that the nasal microbiota may play a key role in the pathogenesis and symptoms of AR and nAR, providing us with new ideas for the treatment of AR and nAR.

## 1 Introduction

The prevalence of Chronic rhinitis (CR) is increasing, and it is reported that more than 500 million people worldwide suffer from the disease (Agnihotri and McGrath, 2019). There are two types of CR: allergic rhinitis (AR) and non-allergic rhinitis (nAR). AR is a Th2 immune response disease caused by IgE-mediated inhalation of allergens, with symptoms such as nasal itching, sneezing, runny nose, and nasal congestion (Sahoyama et al., 2022), which relies on positive skin prick test (SPT) or specific immunoglobulin E (sIgE) teats for diagnosis (Roberts et al., 2016). NAR is a heterogeneous nasal disease with symptoms of nasal itching, sneezing, rhinorrhea, and nasal congestion, but no systemic allergic symptoms, negative sIgE and/or SPT, affecting over 200 million people worldwide (Hellings et al., 2017). At present, the etiologies of AR and NAR are still being further explored (Bousquet et al., 2008).

The study of microbiomes has revealed the importance of microbiota in maintaining human health over the last few decades (Ver Heul et al., 2019). The term “microbiota” refers to all microorganisms that live in the body, including bacteria, fungi, viruses, protozoa, and archaea, among others, and are found in large numbers and varying proportions (Koidl and Untersmayr, 2021). This ratio is dynamic during the first two years of life, after which it tends to balance, and early colonization of this “balanced” and “healthy” microbiota lays the groundwork for lifelong health (Grier et al., 2018). Although most current research has focused on the gut microbiota, the role of microbiota elsewhere in the body in human disease is becoming more recognized (Ver Heul et al., 2019). According to research, microbial diversity can play a positive or negative role in allergic diseases. Staphylococcus nasal colonization was found to be significantly higher in asthmatic patients’ respiratory extracts than in healthy controls, which induced human nasal epithelial cells to release inflammatory factors and aggravated Th2 cell-mediated inflammatory response (Durack et al., 2018). According to research, the abundance of Faecalibacterium in the intestines of asthmatic children is significantly reduced, and the short-chain fatty acids it produces inhibit the accumulation of peripheral Treg cells *via* HDAC, thereby reducing allergic airway diseases (Koidl and Untersmayr, 2021). However, no studies have been conducted to determine whether there are differences in nasal microbiota between nAR and AR patients. As a result, we used high throughput 16S rDNA and metagenomic sequencing to compare the nasal microbiota characteristics of AR, nAR, and healthy controls. The purpose of this study is to determine nasal microbiota distribution differences, specific nasal microbiota and functions related to AR and nAR environmental factors, and functional analysis of key gene pathways and enzymes.

## 2 Material and methods

### 2.1 Research objects and experimental design

From February 2022 to September 2022, patients were consulted in the nasal outpatient department of the Second Affiliated Hospital of Harbin Medical University. 35 cases in the AR group, 35 in the nAR group, and 20 in the healthy control group were selected. A total of 95 subjects were sequenced and analyzed for 16SrDNA of nasal secretions, and 3 were selected in each group. Macro genome sequencing analysis was carried out. This trial was approved by the Ethics Review Committee of the Second Affiliated Hospital of Harbin Medical University (license number: KY2021-360) and registered with the China Clinical Trial Registration Center (registration number ChiCTR2200057919). All subjects and control groups have informed consent, and the case conforms to the ethical norms of the Helsinki Declaration (World MA, 2013).

#### 2.1.1 Incorporate the criteria

The control group: (1) healthy people selected for the physical examination of the Second Affiliated Hospital of Harbin Medical University and the examination results are normal; (2) there is no history of allergies or family allergies; (3) There are no allergy-related symptoms; (4) After a comprehensive physical examination such as allergen testing, no factors that may cause deviation from the results of this test have been found; (5) Voluntary participation in this study.

AR group: (1) Comply with the diagnostic criteria of AR in the Guidelines for the Diagnosis and Treatment of Allergic Rhinitis (2022, Revised Edition) (Subspecialty Group of Rhinology et al., 2022), paroxysmal sneezing, clear water-like runny nose, itch, sneezing, and other symptoms appear 2 or more, and the daily symptoms persist or accumulate more than 1 hour; (2) At least one of the 19 SIgE test result is positive (>=0.35kU/L, household dust mite, house dust, mulberry tree, cat dandruff, dog dandruff, cockroach, amaranth, egg white, milk, shrimp, beef, shellfish, crab, mango, cashew nuts, pineapple, mixed mold, mixed grass, tree pollen); (3) 10 allergens to One less SPT result was positive (house dust mite, dust mite, cockroach, dendritic spores, artemisia annua, birch, cloves, cat hair, dog hair); (4) Total serum IgE positive (>100IU/mL); (5) Voluntary participation in this study.

nAR group: (1) Have different degrees of clinical symptoms such as nasal congestion, runny nose, nasal itching, sneezing, etc.; (2) All SIgE test results are negative (<35kU/L); (3) All SPT test results are negative; (4) Total serum IgE negative (<100IU/mL); (5) Negative for nasal allergen provocation test (NAPT);(6) Participate in this study voluntarily.

#### 2.1.2 Exclusion criteria

The above subjects met the following exclusion criteria: (1) Patients on systemic or topical antibiotics, immune agents, glucocorticoids, and antihistamines within 3 months.; (2) Other related diseases in the nasal cavity: sinusitis, nasal polyps, benign and malignant tumors, nasal boils, carbuncles, intranasal infections, Nose bleeding within 1 month; (3) Other respiratory diseases: chronic obstructive pulmonary disease, asthma, bronchiectasis, tuberculosis, pneumonia, pulmonary heart disease, pulmonary malignant tumors; (4) Hypertension, coronary heart disease, hyperthyroidism, hypothyroidism, liver and kidney dysfunction, blood system diseases, etc.; 5) The patient has a history of mental and neurological diseases; (6) The abnormal examination results of clinical signs before the trial may deviate the results of this trial according to the judgment of the researchers; (7) Patients with nasal irrigation within 2 weeks.

### 2.2 Nasal symptom score table (TNSS) and quality of life questionnaire for nasal conjunctivitis (RQLQ)

Use the total score of nasal symptoms (TNSS) to evaluate the severity of the symptoms. TNSS score: 0 to 3 (0 = asymptomatic; 1 = mild; 2 = moderate; 3 = severe). Mild: no symptoms that cause obvious discomfort; Moderate: Symptoms cause discomfort but do not affect daily life or interfere with sleep; Severe: Symptoms interfere with daily activities and sleep status. Add the points of each symptom, and get a total score is TNSS (Kang et al., 2017). RQLQ is limited by activity restrictions, sleep disorders, non- Eye/nasal symptoms, practical problems, nasal symptoms, eye symptoms, and emotional composition includes a total of 28 items, each dimension is scored separately, and the cumulative total score is the total score of RQLQ (Juniper et al., 1996; Blaiss et al., 2022).

### 2.3 Sample collection

Guide the swab to the lower turbinate area under the nasal endoscope, rotate at least six times until the swab is saturated, remove it, put it in a liquid nitrogen bottle, and refrigerate at -80°C for 15 minutes until DNA is extracted.

### 2.4 16SrDNA and macrogenome sequencing analysis

#### 2.4.1 16SrDNA sequencing analysis

The genomic DNA of the sample is extracted by CTAB or SDS method, then use agarose gel electrophoresis to detect the purity and concentration of DNA, and use sterile water to dilute an appropriate amount of sample to 1ng/μL. Using diluted genomic DNA as a template, select the V3-V4 area and use specific primers with Barcode and high-efficiency high-fidelity enzymes for PCR. The PCR products that passed the test were purified by magnetic beads, quantified by enzyme labeling, and mixed with the same amount of samples according to the concentration of PCR products. After full mixing, use 2% agarose gel electrophoresis to detect the PCR products and construct the library. The constructed library was checked with Qubit and Q-PCR for quantification, and the qualified library will be sequenced.

#### 2.4.2 Macrogenome sequencing analysis

Use 1% agarose gel electrophoresis (AGE) to analyze the purity and integrity of DNA, and use Qubit^®^ dsDNA Assay Kit in Qubit^®^ 2.0 Fluorometer (Life Technologies, CA, USA) to check DNA for quantification. Take an appropriate amount of sample into a centrifuge tube, and dilute the sample with sterile water until the OD value is between 1.8-2.0. Take 1μg genome DNA of the sample and use NEBNext^®^ Ultra, DNA Library Prep Kit for Illumina (NEB, USA) to construct the library. The genomic DNA was randomly sheared into fragments with a length of about 350 bp using Covaris ultrasonic crusher. The obtained fragments were end-repaired, A-tailed, and further ligated with a sequence adapter. The fragments with adapters were PCR amplified, size selected, and purified to construct the library. The constructed library was checked with Qubit2.0 for quantification, diluted to 2ng/ul, and then the insert size of the library was detected with Agilent 2100. After the insert size meets the expectation, the Q-PCR method is used to accurately quantify effective library concentration (effective library concentration is>3nM) to ensure the quality of the library. Quantified libraries will be pooled and sequenced on Illumina PE150 platforms, according to effective library concentration and data amount required.

### 2.5 Statistical analysis

#### 2.5.1 16 SrDNA sequencing statistical analysis

Use the Uparse algorithm to cluster sequences into OTUs and annotate species, use Qiime software to calculate Chao1, Shannon, Simpson and ace indexes, draw diluted curves and species accumulation curves, and analyze differences between Alpha diversity index groups. R software is used to analyze the differences between Beta diversity index groups, including LEfSe analysis, MetaStat analysis, and t.test_bar_plot analysis to compare the differences between groups, calculate the Spearman correlation coefficient values of species and environmental factors and test their significance. Based on species abundance, the correlation coefficient value between each genus is calculated using graphviz-2.38.0 to draw a network diagram, and MeanDecreeAccuracy chose a meaningful genus to build a random forest model.

#### 2.5.2 Statistical analysis of macrogenome sequencing

Use MetaGeneMark for ORF prediction, and use Bowtie2 (Bowtie2.2.4) to compare the Clean Data of each sample to the initial gene catalog for basic information statistics, core-pan gene Analysis, correlation analysis between samples, and gene number Wayne diagram analysis. The sequence extracted from the NR database of NCBI is compared with Unigenes, the LCA algorithm is used to determine the species annotation information, Krona analysis is carried out, and then Metastats and LEfSe analysis are used to find different species between groups. Unigenes were compared with the KEGG database using DIAMOND software for annotated gene number statistics, relative abundance profile display, abundance clustering heat map display, comparative metabolic pathway analysis, and Metastat and LEfSe analysis of functional differences between groups based on abundance at each taxonomic level.

## 3 Results

### 3.1 clinical characteristics of the subjects

The mean age of the AR group was 21.03 ± 9.94, the TNSS score was 10.03 ± 3.67, the RQLQ score was 102.8 ± 29.64; the mean age of the nAR group was 33.73 ± 10.73, the TNSS score 8.77 ± 2.24, RQLQ score 89.67 ± 26.43, mean age of control group: 29.7 ± 12.25, AR group was significantly younger than the nAR group (p<0.001), and TNSS score and RQLQ score did not differ between the two groups (p>0.05) (**Table 1**).

**Table 1:** Clinical characteristics of AR and non-AR subjects.

Group	AR	nAR	B
Subjects (n)	30	30	20
Gender			
Male	14	13	8
Female	16	17	12
Age	21.03 ± 9.94	33.73 ± 10.73	29.7 ± 12.25
SPT (%)	100	0	0
TNSS	10.03 ± 3.67	8.77 ± 2.24	0
Nasal obstruction	3.13 ± 1.07	2.37 ± 1.89	0
Rhinorrhea	2.5 ± 0.97	2.4 ± 1.07	0
Nasal itching	1.87 ± 1.14	1.53 ± 0.9	0
Sneezing	2.53 ± 1.14	2.47 ± 1.07	0
RQLQ	102.8 ± 29.64	89.67 ± 26.43	NA

SPT, skin prick test, TNSS, Total Nasal Symptom Score; RQLQ, Rhinoconjunctivitis Quality of Life Questionnaire, NA: Not available.

### 3.2 16SrDNA sequencing analysis

#### 3.2.1 Comparison of microbial diversity among patients with AR and nAR

**Figure 1:**
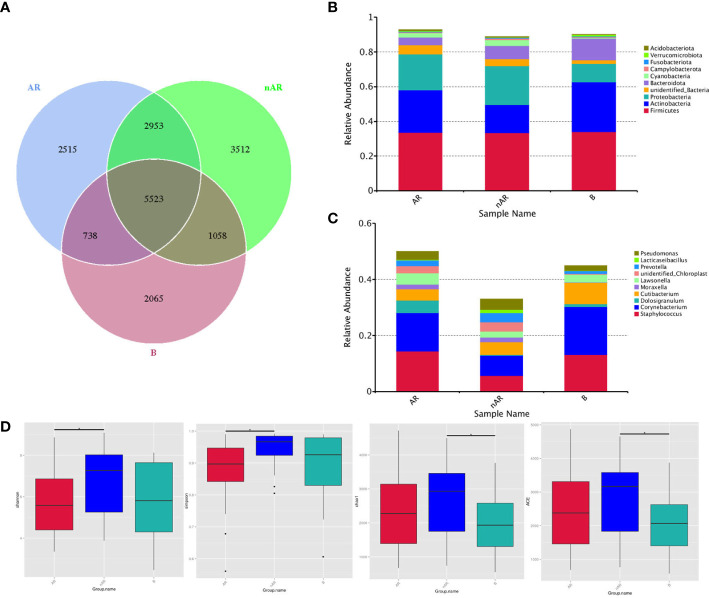
Wayne diagrams were made according to OTU **(A)**. bacterial structure comparisons between AR and non-AR at the phylum **(B)** and genus **(C)** levels, including the top ten genera. bacterial diversity comparisons between AR and non-AR patients. Comparison of bacterial alpha diversity indices, including Shannon, Simpson, Chao1, and ACE **(D)**.

A total of 18,364 OTUs were obtained from the three groups by 16SrDNA assay, with 2515 OTUs specific to the AR group and 3512 OTUs specific to the nAR group, and a total of 8476 OTUs in the two groups. There were fewer specific OTU in the AR group than in the nAR group (**Figure 1A**). A total of 98 bacteria phyla and 1476 genera were detected in the three groups. The common dominant groups were Firmicutes, Actinobacteria, Proteobacteria, Bacteroidota, and cyanobacteria. In AR and nAR groups, the average relative abundance of Actinobacteria was lower than that of the control group (**Figure 1B**), and the average relative abundance of Proteobacteria was higher than that of the control group. Staphylococcus, Corynebacterium, Doloigranulum, Cutibacterium, Moraxella, Lawsonella, Prevotella, Lacticaseibacillus, Pseudomonas, and an unidentified_Chloroplsat were the top 10 genera with the highest relative abundance (**Figure 1C**). There were significant differences in α-diversity Shannon index and Simpson index between AR group and nAR group. The results showed that the microbial diversity of the two groups was significantly different. There was a significant difference in the Chao1 index and ACE index between the nAR group and control group, but there was no significant difference between the AR group and nAR group. The results showed that there were significant differences in microbial abundance between the two groups (**Figure 1D**).

#### 3.2.2 Analysis of microbial β diversity in AR and nAR groups

**Figure 2:**
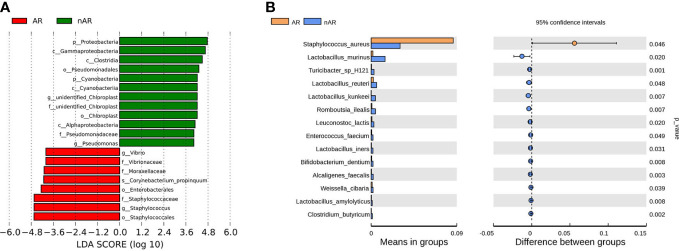
Species difference analysis was performed on the nasal flora of patients with allergic rhinitis and non-allergic rhinitis. lefSe analysis screened for different species with LDA>4 **(A)**, and t.test analysis screened for different species with p<0.05 **(B)**.

The results of LEfSe analysis in β-diversity showed that the relative abundance of Vibrio, Moraxellaceae, and Corynebacterium_propinquum was higher within the AR group, while the relative abundance of Proteobacteria, Gammaproteobacteria, Clostridia, and Pseudomonadales was higher within the nAR group (**Figure 2A**). In the t-test test, the relative abundance of S. aureus was higher within the AR group, and the relative abundance of Lactobacillus murinus, Turicibacter sp H121, Lactobacillus reutrei, Lactobacillus kunkeei, Romboutsia ilealis, Enterococcus faecium, and Lactobacillus iners was higher within the nAR group (**Figure 2B**).

#### 3.2.3 AR group and nAR group have similar network complex patterns

**Figure 3:**
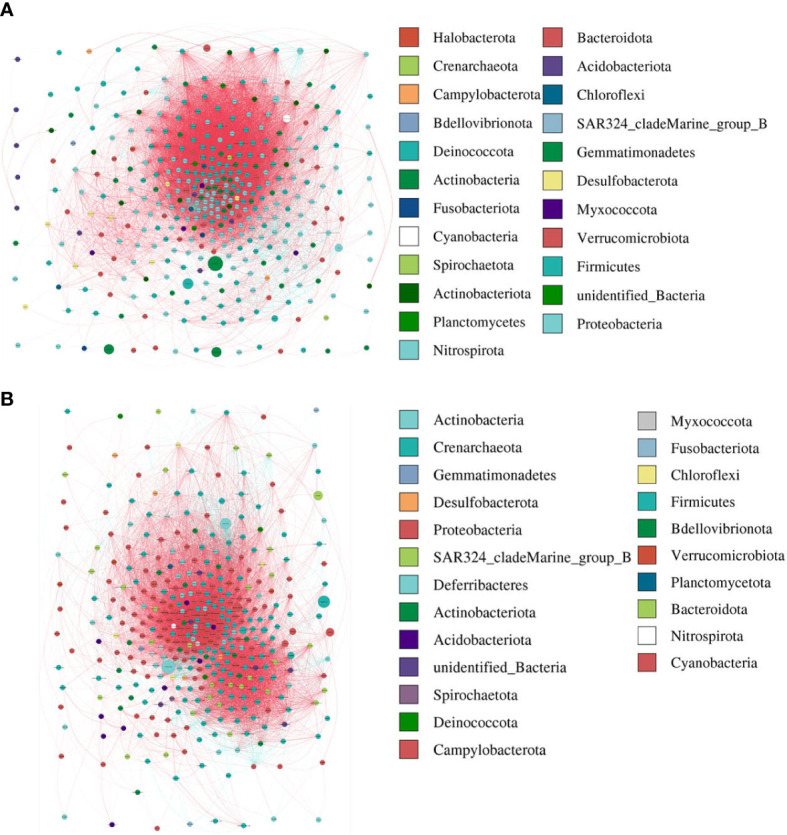
The network analysis between bacterial taxa for AR **(A)** and nAR **(B)** group. Different node color denotes varied phyla taxa and the weighted node size was based on the relative abundance. The weighted edges indicate the correlation coefficient.

The modularity, clustering coefficient, and average degree of the AR group were 0.0956, 0.7359, and 185.54, while in the nAR group, they were 0.319, 0.622, and 155.43. The two groups had similar network complexity patterns, but the main focusing nodes of the two groups were completely different. The focusing nodes of the AR group mainly included Firmicutes, Gemmatimonadetes, Planctomycetes, and Nitrospirota (**Figure 3A**), while the focused nodes in the nAR group were Proteobacteria, Deferribacteres, Verrucomicrobiota (**Figure 3B**).

#### 3.2.4 Environmental factor correlation analysis

The TNSS score was calculated based on the sum of nasal congestion, nasal leakage, nasal itching, and sneezing and represents the severity of AR and nAR symptoms, a higher score means more severe symptoms. the RQLQ score reflects the disease-related quality of life status, therefore, we used spearman rank correlation analysis to correlate age, sex, TNSS and RQLQ scores, EOS, IgE, and bacterial genus correlations were analyzed.

**Figure 4:**
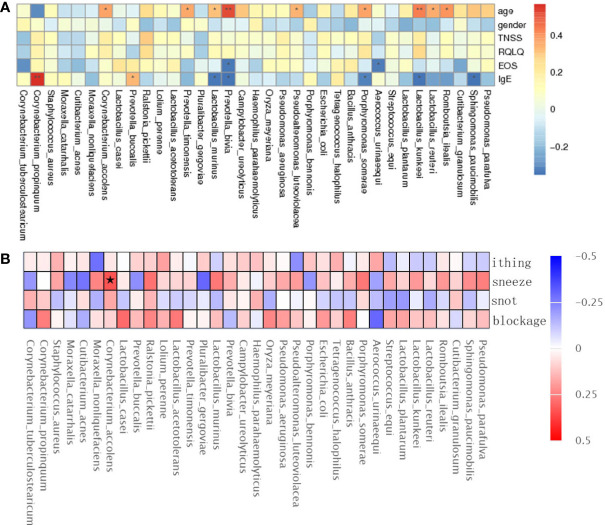
Spearman rank correlation analysis was used to correlate age, sex, TNSS, RQLQ scores, EOS, IgE **(A)**, and TNSS score details **(B)** with bacterial species. Correlation significance,* denotes p < 0.05 and **p < 0.01.

The results showed that Lactobacillus kunkeei, Corynebacterium accolens, Lactobacillus murinus, and Romboutsia ilealis were positively correlated with age; Prevotella bivia and Aerococcus urinaeequi were negatively correlated with eosinophil (EOS); Corynebacterium propinquum and Prevotella buccalis were positively correlated with IgE, and Lactobacillus murinus and Lactobacillus kunkeei were negatively correlated with IgE (**Figure 4A**), and gender, TNSS, and RQLQ scores were not significantly correlated with There was no significant correlation between gender, TNSS and RQLQ scores and flora. When performing spearman analysis of nasal congestion, nasal leakage, nasal itching, and sneezing in TNSS with flora, we found that Corynebacterium accolens was positively correlated with sneezing (**Figure 4B**).

#### 3.2.5 Comparison of microbial communities in patients with moderate and severe symptoms

**Figure 5:**
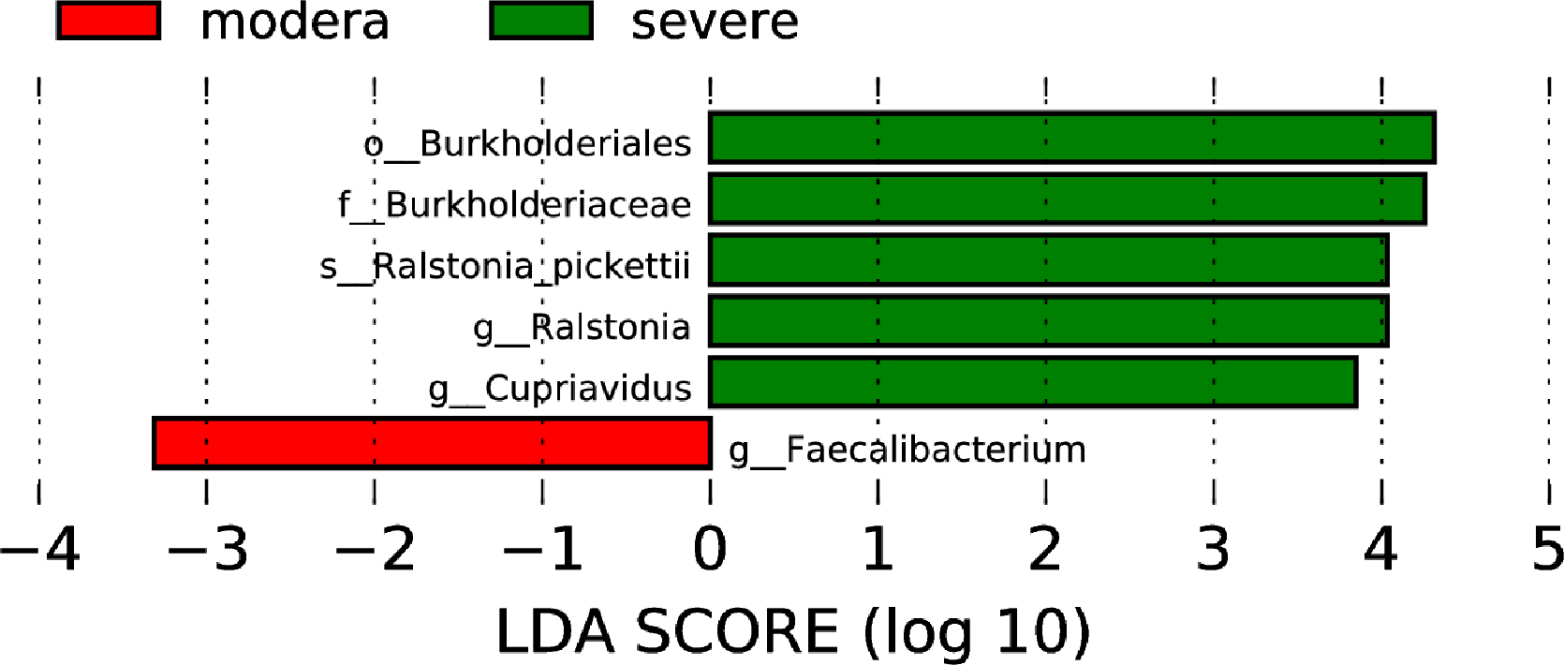
Nasal microbiological differences between moderate and severe patients.

Based on the TNSS score, we divided 60 AR and nAR patients into moderate (score 0-7) and severe (8-16) groups and compared bacterial diversity and community differences to explore the role of bacterial community structure in the progression of AR. Results show that diversity was not significantly different, and LEfSe analysis showed that the mean relative abundance of Faecalibacterium was higher in the moderate group than in the severe group, and the mean relative abundance of Ralstonia pickettii and Cupriavidus was lower than in the severe group, suggesting a role for specific flora in the progression of the disease (**Figure 5**).

#### 3.2.6 A predictive model of nasal microbial distribution for AR and nAR

This is a classical machine learning model based on a classification tree algorithm that provides further support for differentiating AR groups, nAR groups, and control groups. Based on the analysis of OTU features, a random forest prediction model with 5 genera was constructed. Mean Decree Accuracy selected meaningful genera, performed 10-fold cross-validation of the model, plotted working characteristic (ROC) curves, and calculated the area under the curve (AUC) to score the predictive power.

**Figure 6:**
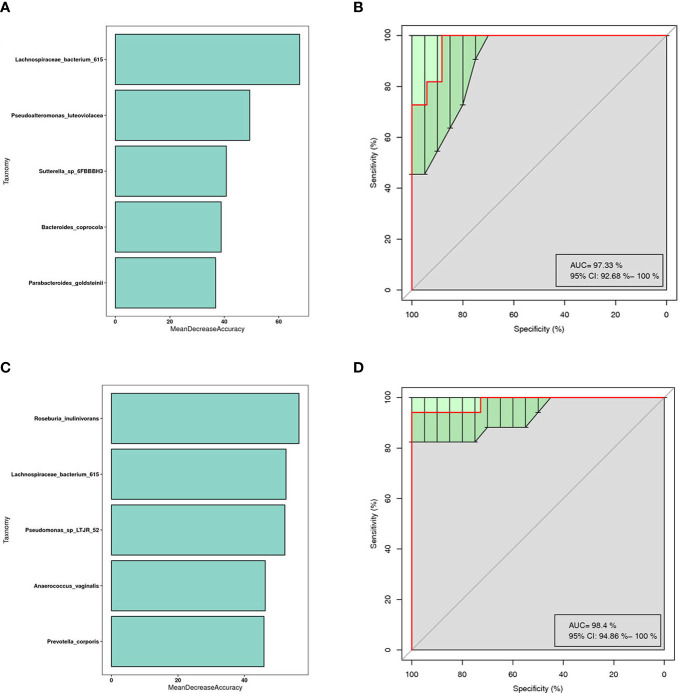
Prediction model of the airway microbiota for AR status based on the species-level relative abundances using random forests.AR **(A)** and nAR **(C)** group of variable importance ranking plots, MeanDecreeAccuracy measures the degree to which the predictive accuracy of the random forest is reduced by changing the values of the variables to random numbers. Higher values indicate more important variables. ROC curves of the AR **(B)** and nAR **(D)** model using 5 discriminatory species.

The results showed that mainly Parabacteroides goldstemii, Lachnospiraceae bacterium 615, Sutterella-SP-6FBBBH3, Pseudoalteromonas luteoviolacea, and Bacteroides coprocola were observed in the models of AR and healthy controls (see **Figure 6A**) with an AUC of 0.9733 (95% CI: 0.926-1.000) (**Figure 6B**); nAR and healthy controls model was observed mainly for Pseudomonas sp-LTJR-52, Lachnospiraceae bacterium-615, Prevotella corporis, Anaeroicoccus vaginalis, and Roseburia inulinivorans (**Figure 6C**) with an AUC of 0.984 (95% CI: 0.949-1.000) (**Figure 6D**), suggesting that the combined nasal biota has the potential to diagnose AR and nAR and could potentially be used as a diagnostic biomarker one, but the random forest model is only a prediction and further trials are needed to validate it.

### 3.3 Metagenomic sequencing analysis

#### 3.3.1 Species composition and variability analysis of AR and nAR groups

**Figure 7:**
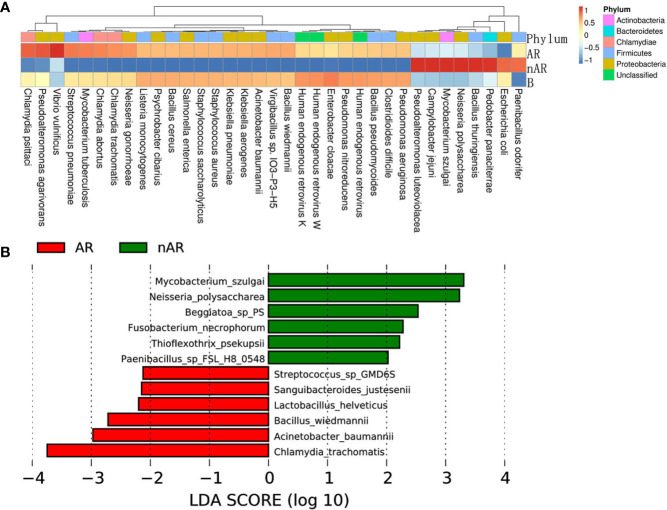
Differences in bacterial composition between AR and nAR in the heat map **(A)** and LEfSe analysis **(B)** by macrogenome sequencing.

By macrogenomics analysis, we obtained a total of 57,140.83 raw data and 56,962.55 post-cleaning data, including 19,771.66 for the AR group and 18,313.67 for the nAR group. the α-diversity analysis did not show positive results, which may be related to the selection of samples. In the species distribution heatmap, we found that the mean relative abundance of Pseudoalteromonas luteoviolacea, E.coli and Dolosigranulum was higher in the nAR group, and the mean relative abundance of Vibrio vulnificus and Streptococcus pneumoniae was higher within the AR group (**Figure 7A**); in the species annotation of the LEfSe analysis, the mean abundance of Neisseria polysaccharea, Mycobacterium szulgai and Thioflexothrix within the nAR group was higher than in the AR group, and Streptococcus sp GMD6S and Acinetobacter baumannii were lower than AR (p < 0.05) (**Figure 7B**).

#### 3.3.2 Differential analysis of specific microbial functions in AR and nAR groups

**Figure 8:**
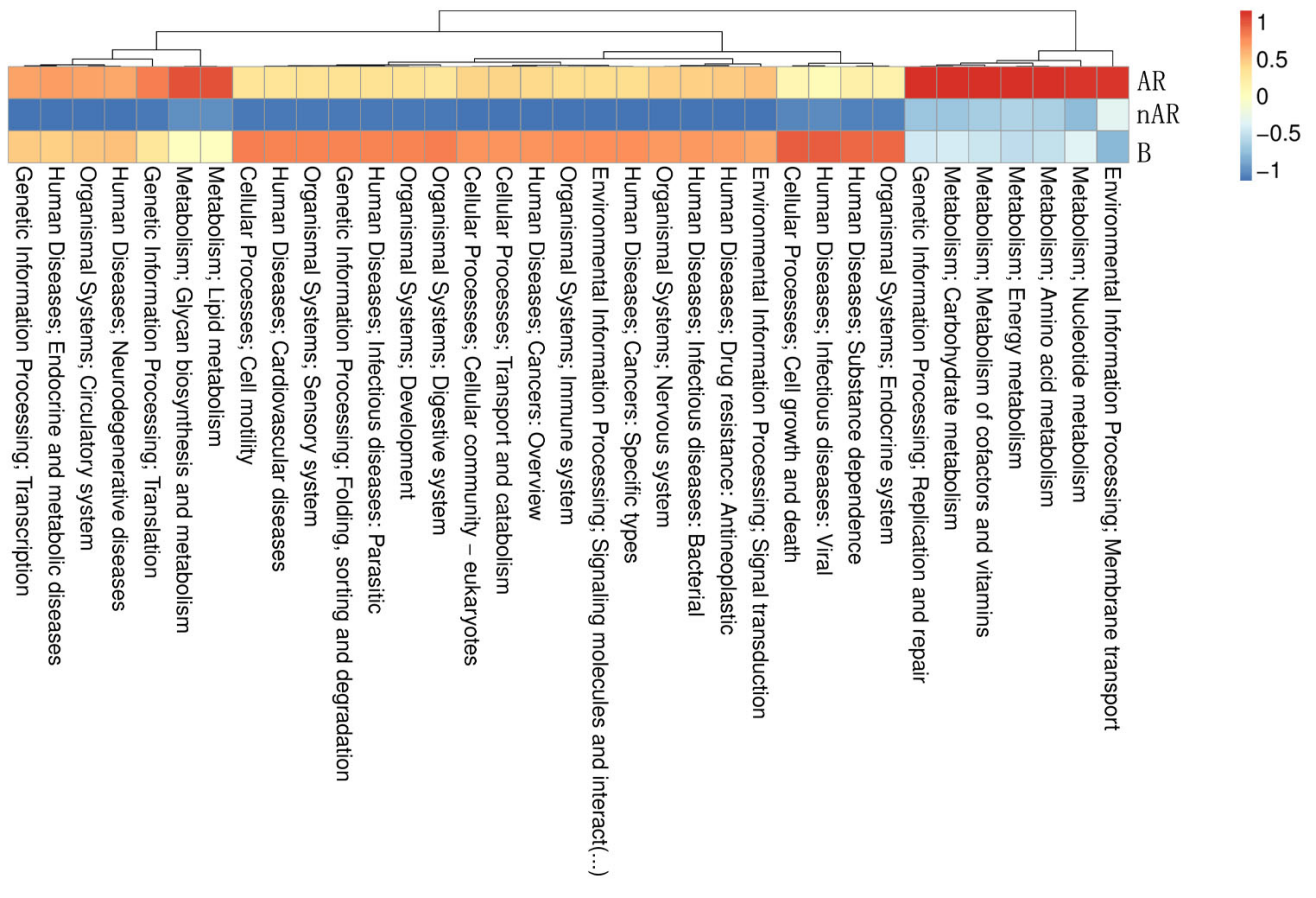
Differential analysis of specific microbial functions in AR and nAR groups compared by KEGG database.

To characterize the different functions of the nasal microbiota, we annotated the KEGG database for macrogenomic functions. Microbial genes for processing of environmental information, metabolism of nucleotides, metabolism of amino acids, metabolic cofactors, and vitamins, metabolism of carbohydrates, metabolism of lipids, biosynthesis, and metabolism of glycans were found to be increased within the AR group; in the nAR group, microbial genes for cell growth and death, processing of environmental information including signal transduction and interaction of signaling molecules, transport, and catabolism, processing of genetic information: folding, sorting and metabolism, microbial genes for biosynthesis and metabolism of glycans were decreased (**Figure 8**).

#### 3.3.3 Pathway detection of microorganisms in AR and nAR groups

By performing pathway assays on the AR and nAR groups, we found that 6-phospho-3-hexuloisomerase, cystathionine beta-synthase, aspartate–ammonia ligase, farnesyl-diphosphate farnesyltransferase, and protein-S-isoprenylcysteine O-methyltransferase were key enzymes specific to the AR group, whereas threonine aldolase, O-ureido-L-serine synthase, tryptophan-7-halogenase, and penicillin acylase were key enzymes specific to the nAR group (**Additional file**).

#### 3.3.4 Differential analysis of carbohydrase in AR and nAR groups

**Figure 9:**
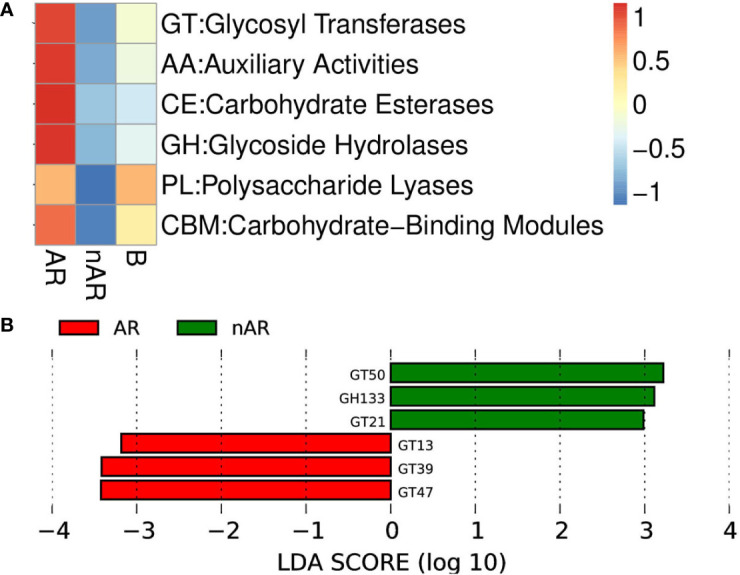
Differences between enzymes within the AR and nAR groups in the heat map **(A)** and LEfSe analysis **(B)** compared by CAZY database.

Using macrogene annotation from the CAZY database, we found that the number of genes for the glycosyltransferase system, carbohydrate esterase system, and glycoside hydrolase system was significantly increased within the AR group and decreased within the nAR group compared with healthy controls (**Figure 9A**), and LefSe analysis showed that within the AR group N-acetylglucosaminyltransferase I, dolichyl-phosphate-mannose—protein, mannosyltransferase, N-acetylglucosaminyl-proteoglycan 4-beta-glucuronosyl transferase significantly increased within the AR group and amylo-alpha-1,6-glucosidase, ceramide glucosyltransferase significantly increased within the nAR group (**Figure 9B**).

## 4 Discussion

The human microbiota is important for the host immune response, metabolism, and disease progression (Blaser et al., 2013). In the present study, we discovered that the nasal microbiota of AR, nAR, and control patients differed significantly in composition and function at multiple microbial levels.

The average relative abundance of vibrio vulnificus and Acinetobacter baumanni increased significantly in the AR group. Vibrio vulnificus is a Gram-negative, halophilic marine bacterium (Blake et al., 1979). It can activate mTOR by recruiting and activating neutrophils, monocytes, and macrophages (Weichhart et al., 2008), activate the NF-kβ signaling pathway *via* TLRs or NLRs, and induce allergic reactions *via* GM-CSF, IFNβ, IL-27, and IL-1β production (Xie et al., 2017). Simultaneously, vibrio vulnificus has an anti-inflammatory effect by inhibiting Kupffer cell proliferation (Blériot et al., 2015), which is consistent with the pathogenesis of AR. According to research, Acinetobacter baumanni activates the Nod-like receptor NLRP3 *via* caspase-1 to promote the release of IL-1β and TNFα from macrophages, thereby inducing asthma (Chai et al., 2022). Simultaneously, Acinetobacter has several virulence factors, including toxins that form pores, and its outer membrane protein A induces dendritic cells to produce ROS, which can activate NLRP3 and promote immune responses such as asthma and allergic rhinitis (Kang et al., 2017).

We observed that Lactobacillus murinus, Lactobacillus iners, and Escherichia coli increased significantly in the nAR patients. Lactobacillus murinus regulates T lymphocyte activity, which helps to maintain intestinal immune homeostasis in a mouse model of colitis (Tang et al., 2015). Lactobacillus murinus and Lactobacillus iners can stimulate macrophage IL-10 release *via* TLR2 signaling, thereby controlling inflammation and preventing immune responses (Hu et al., 2022). Through the inhibition of CD23, Escherichia coli has been shown to promote the transformation of T and B cell subsets to Th1 cells and reduce IgE-mediated allergen presentation (Weise et al., 2011). Previous research found increased numbers of FoxP3+ cells as well as increased production of anti-inflammatory factors TGF-β and IL-10 in the skin of Escherichia coli-treated mice (Cukrowska et al., 2002). Simultaneously, Escherichia coli can increase IgA secretion and inhibit mast cell degranulation to suppress the immune response (Dölle et al., 2014). As a result, we believe that vibrio vulnificus and Acinetobacter baumanni have pathogenic effects in the AR group, whereas in the nAR group, patients did not show Th2-mediated allergic reactions due to the anti-inflammatory effects of Lactobacillus murinus, Lactobacillus iners, and Escherichia coli. Spearman analysis confirmed that IgE was negatively correlated with Lactobacillus murinus and Lacttobacillus kunkeei.

Furthermore, studies have revealed that allergy-induced inflammatory responses occur not only in the IgE/mast cell/basophil axis, but also in macrophages, neutrophils, platelets, endothelial cells, complement initiation, neuropeptide release, and can result in anaphylaxis-like reactions (Cianferoni, 2021). In our study, we discovered that the relative abundance of Proteobacteria and Pseudomonadales increased significantly in the nAR group. Proteobacteria and Pseudomonadales were also found to be significantly enriched in intestinal CD14+CD11c+ macrophage samples from Crohn’s disease patients. Its LPS binds to CD14 and TLR4 to activate the TIRAP-MyD88 pathway, resulting in the release of inflammatory cytokines, and activation of the TLR4 receptor on the endosomal membrane can also produce type 1 interferon *via* the TRAM-TRIF pathway, inducing even more inflammation (Sekido et al., 2020). Human microbiota species are largely similar, but their relative abundance ratio varies with habit anatomic locations and can influence and interact with one another. According to Jakubczyk D et al., intestinal flora imbalance affects the relative abundance of respiratory tract flora (Jakubczyk and Górska, 2021). In our experiments, we obtained similar results. The majority of the bacteria with significant differences in nasal secretions of nAR patients were intestinal resident bacteria, indicating that nasal and intestinal microbes communicate. As a result, we believe that the rise in Proteobacteria and Pseudomonadales is one of the primary causes of nAR.

According to the KEGG database, ICMT is a unique enzyme in the microbiota of AR patients. The TLR-mediated inflammatory response is regulated by ICMT and its substrate Ras protein. Through the MAPK pathway, methylated Ras protein promotes the production of pro-inflammatory factors IL-1β, IL-1α, IL-5, IL-9, IL-17, and TGF-β, which are also common inflammatory factors in AR (Yang et al., 2020). This suggests that we could use ICMT inhibitors to block Ras methylation and thus prevent the occurrence of AR, which will be the goal of our next investigation. Through KEGG functional annotation, we also found that the glycan biosynthesis and metabolism of microbiota increased in AR patients but decreased in nAR patients. Glycans on the cell surface control and participate in cellular interactions and recognition between functional molecules and cells *via* carbohydrate-binding protein (CBP) (Schnaar, 2015). Galectin is the most common CBP, and it promotes immune cell maturation, survival, and activation by binding to target glycans on surface glycoproteins such as TCR, CD45, and CD43 (Rabinovich and Toscano, 2009). GBP can also inhibit T cell activation and promote Th1-to-Th2 transition by inhibiting IFNγ expression and promoting the production of cytokines such as IL-4, IL-5, IL-9, IL-10, and IL-13 (Sanjurjo et al., 2022). Therefore, we believe that glycan biosynthesis and metabolism play a role in the pathogenesis of AR.

We discovered that Lacttobacillus kunkeei was positively correlated with age using correlation analysis. In this study, the average age of onset in the AR group was 21.03 ± 9.94 years, while it was 33.73 ± 10.73 years in the nAR group. The relative abundance of Lacttobacillus kunkeei increased in the nAR group, implying that increased pathogenicity manifested by microbiota changes may be age-related. Previous studies have shown an association between Lactobacillus and age, with increased abundance with age (Sanjurjo et al., 2022) and that Lactobacillus increases levels of the anti-inflammatory cytokine IL-10 and decreases levels of the pro-inflammatory cytokines TNF-α and ROS (Hu et al., 2022). Therefore, we suggest that the gradual increase of Lactobacillus kunckii with age limits the occurrence of allergic reactions. Using the TNSS score, we discovered a significant difference in the composition of the microbiota between the moderate and severe disease groups. The moderate group had a higher average relative abundance of Faecalibacterium, which could be related to its anti-inflammatory effect (Martín et al., 2017). Faecalibacterium can secrete MAM, which interacts with the ZO-1 protein to maintain the integrity of the tight junction complex by connecting cohesin, occludin, and cytoskeleton protein, thereby preventing systemic complications caused by pathogens and bacterial toxins entering the blood (Xu et al., 2020). As a result, Faecalibacterium may be beneficial in the process of CR disease.

Previous research has shown that random forest analysis can predict the occurrence of AR (Yuan et al., 2022). Our findings suggest that combining the detection of Parabacteroides goldstemii, Sutterella-SP-6FBBBBH3, Pseudoalteromonas luteoviolacea, Lachnospiraceae bacterium-615, and Bacteroides coprocola can be used as a diagnostic biomarker for AR, whereas Pseudomonas-SP-LTJR-52, Prevotella corporis, Anaerococcus vaginalis, Lachnospiraceae bacterium-615, and Roseburia inulinivorans can be used for nAR.

Currently, there is growing interest in the application of probiotics to modulate microecological balance in the treatment of AR, defined by the World Health Organization as living microorganisms that, when administered in adequate amounts, provide health benefits to the host. This beneficial effect was initially thought to stem from improved gut microbial balance, but there is now substantial evidence that probiotics can also provide benefits by modulating immune function (Cortes-Perez et al., 2021). In animal models, probiotic supplementation can protect the organism from spontaneous and chemically induced colitis by downregulating inflammatory cytokines or inducing regulatory mechanisms in a strain-specific manner; in animal models of allergen sensitization and murine models of asthma and allergic rhinitis, oral probiotics can reduce allergen-specific IgE production in an allergen-dependent manner by modulating systemic cytokine production (Ballan et al., 2020), Ahmed et al. demonstrated the same effect of cetirizine and Lactobacillus casei in children under 5 years of age with perennial AR. Children given daily intake of Lactobacillus casei (2 × 10^9^ CFU) or cetirizine (2.5-5 mg) showed significant improvement in baseline AR symptoms in more than 95% of participants after 6 weeks of intervention (Ahmed et al., 2019). This validates our results that microbial homeostasis imbalance plays a key role in the development of disease, that regulating microbial diversity will improve the symptoms of AR, and that the prospect of probiotic applications needs to be explored in more depth, which provides a direction for our future research.

## 5 Conclusion

In conclusion, this study establishes nasal microecological regulation as a potential therapeutic target for AR and nAR. We discovered that AR patients differed significantly from nAR patients and healthy controls in terms of nasal bacterial α and β diversity. AR is closely associated with an increase in the relative abundance of vibrio vulnificus and Acinetobacter baumanni, and an increase in the relative abundance of Lactobacillus iners, Lactobacillus murinus, and Escherichia coli may also be a key factor in the occurrence of nAR. The anti-inflammatory effect of probiotics such as Lacttobacillus kunkeei and Escherichia iners and the antagonism of ICMT could be a future treatment strategy for AR. Glycan biosynthesis and metabolism may play a role in the pathogenesis of AR, which will be investigated further in the following step. The joint detection of microbiota based on random forest results may also provide us with new ideas for future AR and nAR diagnosis.

## Data availability statement

The data presented in the study are deposited in the NCBI repository, accession number PRJNA936719.

## Ethics statement

The studies involving human participants were reviewed and approved by China Clinical Trial Registration Center, CHINA. The patients/participants provided their written informed consent to participate in this study.

## Author contributions

JW and YS conceived and designed the research. YC, NW, and QM conducted the experiments. JL and ZX analyzed the data. YC and QL wrote and edited the manuscript. All authors contributed to the article and approved the submitted version.
