# Supplementary material for: Microbial characterization of the nasal cavity in patients with allergic rhinitis and non-allergic rhinitis
Source: Front Cell Infect Microbiol. 2023 Apr 25;13:1166389. doi: 10.3389/fcimb.2023.1166389 (PMC10166850; doi:10.3389/fcimb.2023.1166389)
Supplement: Supplementary file 1 [file DataSheet_1.docx]

Supplementary Material

Microbial characterization of the nasal cavity in patients with allergic rhinitis and non-allergic rhinitis

Yanlu Che^1^, Nan Wang^1^, Qianzi Ma^1^, Junjie Liu^1^, Zhaonan Xu^1^, Qiuying Li^1^, Jingting Wang^1^*† and Yanan Sun^1^*†

*** Correspondence:**Jingting Wang1*† and Yanan Sun1*†

Corresponding Author: 5433@hrbmu.edu.cn;76202920@qq.com

## 1 Supplementary Figures


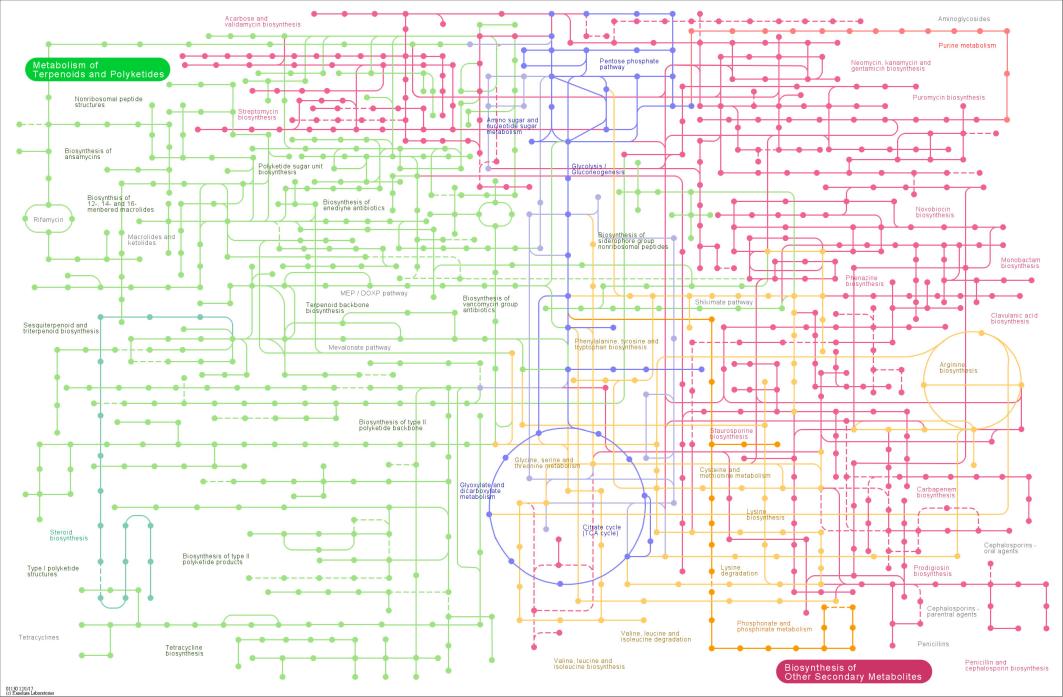


**Supplementary Figure 1.** Pathway detection of microorganisms in AR and nAR groups.
